# Impact of Food Preparation Video Exposure on Online Nutrition Education in Women, Infants, and Children (WIC) Program Participants: Retrospective Study

**DOI:** 10.2196/12508

**Published:** 2019-01-23

**Authors:** Robert J Bensley, John J Brusk

**Affiliations:** 1 School of Interdisciplinary Health Programs Western Michigan University Kalamazoo, MI United States

**Keywords:** internet, online video, WIC, engagement

## Abstract

**Background:**

The impact of integrating video into health education delivery has been extensively investigated; however, the effect of integrating video on a learner’s subsequent performance in an online educational setting is rarely reported. Results of the relationship between the learner’s online video viewing and subsequent progression toward health behavior change in a self-directed online educational session are lacking.

**Objective:**

This study aimed to determine the relationship between viewing a Health eKitchen online video and key engagement performance indicators associated with online nutrition education for women, infants, and children (WIC).

**Methods:**

This study involved a retrospective cohort of users grouped on the basis of whether Health eKitchen exposure occurred before or after completing a nutrition education lesson. A two-sample test for equality of proportions was performed to test the difference in the likelihood of progression between the groups overall and when stratified by lesson type, which was defined by whether the lesson focused on food preparation. Welch two-sample *t* tests were performed to test the difference in average link depth and duration of use between groups overall and stratified by lesson type. Logistic regression was conducted to validate the impact of video viewing prior to lesson completion while controlling for lesson type and factors known to be associated with WIC key performance indicators.

**Results:**

A greater stage of change progression was observed for both food preparation (χ^2^=12.6, *P*<.001) and non-food preparation (χ^2^=62.8, *P*<.001) lessons among early stage users who had viewed a Health eKitchen video before completing a lesson. Time spent viewing educational learning resource links within the lesson was also significantly longer for both food preparation (*t*=7.8, *P*<.001) and non-food preparation (*t*=2.5, *P*=.01) lessons. Logistic regression analysis corroborated these results while controlling for known confounding factors. The odds of user progression were nearly three times greater among those who viewed a Health eKitchen video prior to lesson completion (odds ratio=2.61; 95% CI=2.08-3.29). Type of lesson (food vs non-food preparation) was the strongest predictor of progression odds (odds ratio=3.12; 95% CI=2.47-3.95).

**Conclusions:**

User access to a Health eKitchen video prior to completion of an online educational session had a significant impact on achieving lesson goals, regardless of the food preparation focus. This observation suggests the potential benefit of providing an application-oriented video at the onset of online nutrition education lessons.

## Introduction

The federally funded Special Supplemental Nutrition Program for Women, Infants, and Children (WIC) provides qualified program participants who are at nutrition risk due to insufficient financial access to nutritious foods to supplement diets, information on healthy eating, and referrals to health care. Many positive health outcomes have been associated with the program, including improved diet, infant feeding practices, and preconceptual nutrition status [1]. Wichealth.org [2] is an online educational framework in which clients engage to meet the educational requirements in order to be eligible for the WIC program. People who complete wichealth.org lessons demonstrate very high rates of positive movement with belief in their ability to engage in various health-promoting nutritional and physical activity behaviors [3,4]. Over the last decade, many enhancements have been made to wichealth.org to provide continuous improvement with respect to various key performance indicators. These enhancements indicate the value of education for the client, including both engagement extent and duration, as well as the subsequent impact to the client’s current readiness to change behavior. One of these enhancements was the Health eKitchen curated library of existing internet-based food preparation and cooking videos available to users of wichealth.org. Videos consist of short segments (eg, 2-3 minutes in length) that demonstrate how to prepare and cook recipes using specific foods associated with the WIC food package. A recent review of Health eKitchen usage patterns suggested a positive relationship between video access and wichealth.org user lesson progression.

The impact of integrating video into health education delivery has been extensively investigated to determine whether learning objectives can be achieved more effectively and with greater learner satisfaction than interventions that do not use any technology [5-10]. This is also true for other educational disciplines that do not focus on health [11-14]. Typical investigations concerning the use of video, whether integrated into a face-to-face or online educational setting or not, aim at comparing the use of one or more modalities on the learning outcome of interest [15-17]. However, the effect of integrating video on a learner’s subsequent performance in an online educational setting is scarcely reported [18]. Further, to our knowledge, no study has specifically reported on the relationship between learner online video viewing and the learner’s subsequent progression toward health behavior change in a self-directed online educational session. Studies that focused on the effect of video use within the context of an online educational setting tended to focus on user satisfaction and knowledge recall, where online learner satisfaction has been demonstrated to be strongly associated with learner engagement in the educational content [19-21]. Furthermore, inclusion of videos can increase learner motivation to learn and subsequent engagement with online course content [16].

Typical studies concerning the impact of video use in an online learning environment focus on comparing various educational modalities that include video with those that do not or those that have varying levels of interactive video integrated [14,16,22]. Choi and Johnson conducted similar work, wherein various online learning environments that included or did not include the use of video instruction were compared [23]. Most reports identified a significant improvement in learners’ motivation when online videos were integrated. Increase in online learner engagement through the use of videos has also been demonstrated physiologically: Using electrodermal activity and heart rate measurements, van Bruinessen and colleagues showed that a moderate level of increased arousal is ideal to increase learning capacity in an online self-help intervention [18].

The purpose of this study was to determine the relationship between viewing of a Health eKitchen online video and key engagement performance indicators associated with WIC online nutrition education.

## Methods

### Participants

The sample used for this study was derived from WIC clients across 21 US states, who completed a wichealth.org lesson during a 34-month period in the government’s 2014-2016 fiscal years. Participating subjects chose to complete a wichealth.org lesson as a means of meeting secondary contact requirements associated with the WIC program. Data-collection protocols using wichealth.org were approved for use by the Western Michigan University’s Human Subjects Institutional Review Board. Online informed consent was provided prior to completion of the online survey.

### Data Collection

Data utilized in this study were collected from 4460 wichealth.org uses from the system database for WIC clients who started an English-based lesson in one of the first three stages of the Transtheoretical Model (ie, precontemplation, contemplation, and preparation), completed the lesson, and viewed at least one Health eKitchen video during their session. Only the individuals who began a lesson in the early stages of readiness to change, not those in the active change stages, could have measurable progression at the time of lesson completion.

All lessons were completed using the wichealth.org website application. Data consisted of six system-collected measures (links viewed, link view time, device type, beginning stage of change, ending stage of change, and lessons completed), four profile items (ethnicity, race, pregnancy status, and age), and time at which the Health eKitchen video was viewed. The staging algorithms used to identify the beginning and ending stages were based on criteria previously used to determine stages of change and progression, which have been described in detail elsewhere [3,4].

### Statistical Analysis

Binary progression—irrespective of whether a subject advanced in stage of change intent—was the dependent variable used. The primary independent variable of interest was subject exposure to a Health eKitchen video prior to completion of a wichealth.org lesson. Over the past decade, program evaluations conducted every 6 months demonstrated the importance of other variables currently collected from users of wichealth.org. These include user demographic characteristics of race, ethnicity, pregnancy status, and age as well as system access characteristics including device type (fixed, such as a desktop or laptop, vs mobile), lesson type (feeding behavior focus vs other), number of internet resource links accessed (depth), and duration of all resource links used. Each of these variables was considered a possible effect modifier or confounder of the relationship of interest. Depth and duration of link use are key performance indicators for wichealth.org previously demonstrated to be strongly associated with users’ stage of change progression [24]. Lesson type was also assigned based on whether the wichealth.org lesson content focused on feeding behaviors that would be relevant to Health eKitchen videos about food preparation and recipes. Lesson type was included in the model to determine whether the impact of a video view on a lesson is associated with whether the lesson addresses content related to food preparation and recipes.

In order to control for motivation bias potentially confounding the positive relationship between lesson engagement and viewing a Health eKitchen video prior to wichealth.org lesson completion, users who chose to view a Health eKitchen video after completing a wichealth.org lesson were used as the comparison group. Similar to users who viewed a Health eKitchen video prior to their lesson, these individuals were motivated to view a video, but they viewed the video after their lesson. As users access Health eKitchen by their own choice and motivation, it is likely they are motivated to be engaged to a greater extent and subsequently more likely to progress in the stage of readiness change compared to users that opt not to access Health eKitchen at all.

A two-sample test for equality of proportions was performed to test the difference in the likelihood of progression between comparison groups. Likewise, this difference was tested for each of the covariates: Welch two-sample *t* tests were performed to test whether link depth and duration were associated with user progression and each of the covariates. The Welch *t* test adjusts the number of degrees of freedom when the variances are not equal between groups. A logistic regression was conducted to evaluate the odds of progression for users who viewed a Health eKitchen video prior to subsequent completion of a wichealth.org lesson compared to those who did not. All data were analyzed using the R statistical package [25].

## Results

Approximately half (2301/4460, 51.59%) of the subjects included in this investigation reported that they were of Latino ethnicity, and 58.5% of subjects belonged to a race other than white. Most (4206/4460, 94.30%) subjects were mothers of the child who was receiving WIC benefits. In addition, 2655 of the subjects (59.53%) completed the lessons using a fixed device. As expected, device type and lesson type were both significantly associated with the outcome of interest or at least one of the wichealth.org key performance indicators (*P*<.001; Table 1). Further, age group was associated with the outcome of interest and both wichealth.org key performance indicators presented. None of the other demographic variables were significantly associated with progression, link use depth, or link use duration (Table 1).

The wichealth.org stage of change progression (one or more stages) was significantly associated with whether a user accessed a Health eKitchen video prior to their lesson (χ^2^=127.2, *P*<.001; Table 2). Further, the average time spent by users on links during the lesson was significantly more for users who viewed a Health eKitchen video prior to the lesson (*t*=8.2, *P*<.001). In addition, both food preparation (χ^2^=12.6, *P*<.001) and non-food preparation (χ^2^=62.8, *P*<.001) lessons demonstrated significantly greater progression among early stage of change subjects who viewed a Health eKitchen video prior to the lesson. Similarly, the average time spent was significantly longer for both food preparation (*t*=7.8, *P*<.001) and non-food preparation (*t*=2.5, *P*=.01) lessons among users who viewed a Health eKitchen video prior to the lesson.

Only predictors significantly associated with the odds of stage progression were included in the model (Table 3). Although age group was associated with the outcome of interest, it was not a significant contributor to the model and was therefore not included. Viewing of a Health eKitchen video prior to completion of a lesson was associated with nearly three times the odds of progression as that for subjects who did not view a Health eKitchen video until after lesson completion (odds ratio=2.61; 95% CI=2.08-3.29). Lesson type was the strongest predictor of progression odds (odds ratio=3.12; 95% CI=2.47-3.95).

**Table 1:** Characteristics of early beginning-stage users who accessed Health eKitchen.

Demographic		Early state users (N=4460)	% Progression in stage of change	Average link views	Average link minutes
**Device type**					
	Fixed	2655	91.1	2.8^a^	5.7^a^
	Mobile	1805	91.3	1.1	1.7
**Lesson type**					
	Non-food preparation	1557	81.5	1.9	3.2
	Food preparation	2903	96.3^a^	2.2	4.6^a^
**Race**					
	White	1853	91.3	2.2	4.0
	Non-white	2607	91.1	2.1	4.1
**Latino ethnicity**					
	No	2159	89.7	2.2	4.0
	Yes	2301	92.5	2.0	4.1
**Pregnancy status**					
	No	3694	91.4	2.1	4.1
	Yes	767	89.8	2.2	4.1
**User age group**					
	<31 years	2404	90.1	2.0	3.5
	≥31 years	1972	92.5^a^	2.3^a^	4.8^a^

^a^*P*<.001.

**Table 2:** Key performance indicators according to Health eKitchen video viewing.

Health eKitchen video viewed prior to lesson		Early state users (N=4460)	% Progression in stage of change	Average link views	Average link minutes
**Overall**					
	No	913	81.7	2.08	2.69
	Yes	3547	93.6^a^	2.14	4.45^a^
**Non-food preparation**		1557	81.5	1.9	3.2
	No	470	71.7	1.9	2.5
	Yes	1087	85.7^a^	1.9	3.5^b^
**Food preparation**		2903	96.3	2.2	4.6
	No	443	92.3	2.3	2.9
	Yes	2460	97.1^a^	2.2	4.9^a^

^a^*P*<.001.

^b^*P*=.01.

**Table 3:** Results of independent samples study group logistic regression model.

Predictor of model feature	Estimate *B*	SE *B*	*z*	Pr(>|*z*|)	Odds ratio	95% CI
Intercept	0.39	0.12	3.24	0.00121^a^	1.47	1.17-1.87
Health eKitchen view prior to the lesson	0.96	0.12	8.22	0.00000^b^	2.61	2.08-3.29
Lesson type	1.14	0.12	9.53	0.00000^b^	3.12	2.47-3.95
Device type	0.49	0.12	4.07	0.00005^b^	1.63	1.29-2.07
Link views	0.28	0.04	6.30	0.00000^b^	1.32	1.22-1.43
Link minutes	0.05	0.02	2.59	0.00967^a^	1.05	1.01-1.09

^a^*P*<.01.

^b^*P*<.001.

## Discussion

### Principal Findings

Video use has been well demonstrated as a valuable and impactful tool in the online educational setting. This study evaluated the effect of video viewing on intent to change parent-child feeding behavior when the video was viewed before beginning the educational lesson. Potential for confounding caused by motivation or volunteer bias associated with learners who chose to view a Health eKitchen video prior to their lesson was minimized, as the comparison group also chose to view Health eKitchen videos but did so after completion of their educational session.

The results of this study are important, because even though online nutrition education programs have been found to have an impact on various aspects associated with nutrition behaviors among WIC clients, including belief in ability and intent to positively impact parent-child feeding, knowledge and behaviors surrounding eating breakfast, and reduction in salt intake [3-5,26,27], the specific effect of video viewing on achieving online educational objectives when viewed by learners before engaging in a lesson of interest is not well reported in the literature, especially in the WIC setting. To our knowledge, only one published study deemed the use of a training video on how to access a WIC online nutrition education program as helpful in reducing challenges with online access [5]. However, examining the usefulness of instructional video on tasks associated with logging in and negotiating a website is very different from determining the relationship between video viewing and key performance indicator engagement, which was the basis of the current study.

Findings from this investigation suggest that early stage wichealth.org users were more successful in progressing in stage of behavior change intent when the session focusing on that behavior was preceded by a Health eKitchen video view as compared to when it was followed by video viewing while controlling for other key predictors of progression. It was expected that if an effect was identified, it would be limited to lessons focusing on meal-preparation behaviors, as Health eKitchen videos would mostly be relevant to such behaviors, rather than lessons focusing on, for example, behaviors such as physical activity or dental hygiene. However, users of non-meal preparation lessons benefited to a similar extent as users of meal preparation lessons when they used Health eKitchen before completing their lesson, suggesting that the content of the video may be less important than the arousal or level of engagement that may be incited in the learner following video viewing. A previous study on the use of video and its ability to enable deep learning that touched on the subject reported that the relevance of the video with a learning objective should be carefully considered and clear to the user [28]. In contrast, the current study indicated that the use of video may have a positive impact regardless of whether it is specifically related to a behavior change focus. Here, the degree of relevance may be important. In addition, the fact that the Health eKitchen videos are related to personal health choices may be relevant enough to stimulate the user’s motivation and interest in learning more about different, yet related, topics.

### Limitations

Subjects chose whether they wanted to use wichealth.org as the method for completing secondary contact requirements, which limits the ability to generalize the results of this study to the entire WIC population. In addition, Health eKitchen is a separate feature that does not qualify for secondary contact credit. As such, the two groups compared in this study may differ in how they approach educational learning. Nonetheless, the use of video prior to initiating an online learning session appears to be an important strategy to employ in e-learning environment designs in order to maximize user engagement and subsequent achievement of learning objectives.

### Recommendations

We recommend that a more rigorous study be conducted to confirm our findings and allow generalization of the results to a broad audience of WIC participants. Additional studies could focus on introducing video learning in other online education and behavior change programs used by WIC to determine if our findings are applicable to any online learning approach with WIC clients or specific to the Health eKitchen feature available within the wichealth.org nutrition education platform. The use of video prior to the use of other traditional educational formats available within WIC clinics, such as information malls and one-on-one nutrition counseling, could be explored as a means for potentially impacting client engagement. Many clinics provide kiosks or looping videos in clinic waiting areas where nutrition-related videos could be shown. Although not the same as an online format, the implication that the use of video may, to some degree, influence client engagement warrants exploration of this method of learning.

### Conclusions

This study is useful to nutritionists and WIC staff for developing or adopting online nutrition education and behavior change interventions, especially for WIC populations. This study showed that viewing a video prior to lesson completion was positively related to subject engagement in the online educational session.

## Abbreviations

WIC: Women, Infants, and Children
